# Fucoxanthin Bioproduction from Marine Diatoms: Regulatory Strategies and Industrial Potential of *Phaeodactylum tricornutum* and *Odontella aurita*—A Review

**DOI:** 10.3390/md24080282

**Published:** 2026-08-16

**Authors:** Man Zhang, Haoyu Li, Feichao Du, Yuhua Li, Haixing Li

**Affiliations:** 1 College of Fisheries, Henan Normal University, Xinxiang 453007, China; 17732879037@163.com (H.L.); dufeichao@htu.edu.cn (F.D.); 13332857648@163.com (Y.L.); 17530865359@163.com (H.L.); 2 Engineering Technology Research Center of Henan Province for Aquatic Animal Cultivation, Henan Normal University, Xinxiang 453007, China; 3 Key Laboratory of Yellow River and Huai River Water Environment and Pollution Control, Ministry of Education, Henan Normal University, Xinxiang 453007, China

**Keywords:** fucoxanthin, volumetric productivity, marine natural products, sustainable bioproduction, accumulation regulation

## Abstract

Fucoxanthin is a high-value marine xanthophyll with a unique epoxy-allene structure, predominantly produced by brown macroalgae and marine diatoms, and additionally reported in other heterokont lineages including chrysophytes and certain haptophytes. As a core light-harvesting pigment, it exhibits multiple bioactivities including lipid-lowering, antioxidant, and anti-inflammatory effects, with broad applications in functional foods, dietary supplements, and pharmaceuticals. Currently, over 70% of commercial fucoxanthin is extracted from brown macroalgae such as *Saccharina japonica* and *Undaria pinnatifida*. However, due to low endogenous pigment content and significant extraction losses, the industrial yield is only 0.05–0.1% of dry weight, failing to meet the growing downstream demand for stable high-purity supply. Marine diatoms, characterized by short growth cycles, high pigment content, and controllable culture conditions, have emerged as a core direction for industrial upgrading. Among them, *Phaeodactylum tricornutum* and *Odontella aurita* are the two most systematically studied high-yield strains with outstanding potential. This review adopts volumetric productivity (mg/(L·d)) as the core evaluation metric, which integrates biomass concentration, pigment content, and production cycle, and reflects industrial efficiency more reliably than single intracellular content data. We summarize high-yield induction methods from the dimensions of nutrient regulation, light optimization, exogenous induction, and strain improvement, and systematically compare downstream processing, safety profiles, and regulatory status between the two diatoms. Furthermore, we evaluate multi-product co-production potential and scale-up performance from a techno-economic perspective. This review provides a side-by-side benchmark of these two marine diatoms, offering a data-driven reference for process development and industrial deployment of microalgae-derived fucoxanthin.

## 1. Introduction

Marine natural products have long been recognized as an underexploited reservoir of bioactive molecules with prominent nutraceutical and pharmaceutical values. Among marine-derived functional ingredients, carotenoids represent one of the fastest-growing categories due to their high safety profile and multifunctional biological activities. Fucoxanthin (Fx) is an oxygenated xanthophyll carotenoid with a unique epoxy structure and allene bond. It serves as a core functional pigment in the light-harvesting complexes of photosynthetic organisms including marine diatoms and brown macroalgae.

Modern pharmacological studies have confirmed that fucoxanthin exerts significant activities in lipid metabolism regulation, antioxidant defense, anti-inflammation, anti-tumor effects, insulin resistance improvement and neuroprotection. Recent mechanistic investigations have demonstrated that fucoxanthin promotes adipose browning and fatty acid β-oxidation via the AMPK/PGC-1α signaling pathway [1,2], and exerts neuroprotective effects by suppressing microglial overactivation and reducing reactive oxygen species (ROS) accumulation [3]. These findings have further solidified its position as a next-generation high-value marine natural product with broad applications in functional foods, dietary supplements, pharmaceutical raw materials and cosmetic active ingredients.

Currently, the global commercial fucoxanthin supply is still dominated by extraction from brown macroalgae, with raw materials mainly from wild or farmed brown algae such as *Saccharina japonica*, *Undaria pinnatifida* and *Sargassum fusiforme* [4]. This production route faces dual bottlenecks in production capacity and product quality. First, the endogenous fucoxanthin content in brown macroalgae is naturally low, and significant degradation occurs during extraction and purification. The actual yield in industrial scale production is only 0.05–0.1% of dry algal weight [5]. It is necessary to distinguish three related but distinct parameters throughout the production chain: natural intracellular fucoxanthin concentration refers to the inherent pigment content in raw algal biomass, determined by species and cultivation conditions; extraction and purification recovery represents the process efficiency of downstream processing, reflecting the proportion of target pigment retained after extraction and refining steps; final commercial production yield describes the actual yield of qualified end product after the full industrial chain, integrating all losses from harvesting, extraction, purification and quality screening. This huge gap between intracellular content and final commercial yield means tons of dry raw materials are required to produce 1 kg of high-purity product, resulting in huge raw material consumption and high production costs. Second, macroalgae harvesting is highly seasonal: fucoxanthin content in raw materials varies greatly across seasons and is significantly affected by marine climate and environmental changes. The supply chain is further vulnerable to climate change, ocean acidification and marine pollution, exacerbating quality fluctuation and supply instability [4,6]. More prominently, there is notable conceptual confusion and quality differentiation in the raw material market: fucoxanthin and fucoidan often appear together in brown algae extract products, and many products labeled ‘fucoxanthin’ actually take fucoidan as the main functional ingredient. The effective supply of truly high-purity (HPLC ≥ 90%) food-grade and pharmaceutical-grade fucoxanthin is very limited, and there is no unified global production statistics caliber [7].

Marine diatoms, with short growth cycles (7–16 days), high intracellular pigment content, controllable culture conditions and year-round continuous closed production, have become the core technical direction for the industrial upgrading of fucoxanthin production. Among dozens of reported fucoxanthin-producing diatoms, *P. tricornutum*, as a model diatom with fully sequenced genome, has clear genetic background and the most systematic research on regulation technologies [8]. *O. aurita* combines high biomass and high pigment accumulation capacity, and can co-produce eicosapentaenoic acid (EPA) and chrysolaminarin simultaneously, with outstanding comprehensive economic benefits. The two strains are currently the fastest advancing high-yield candidates for industrialization [9].

In terms of food regulatory approval, both strains have made phased regulatory progress in major markets including the EU, the US and Japan [10]. In the US, which adopts a dietary supplement notification system, fucoxanthin extract derived from *P. tricornutum* has completed the FDA New Dietary Ingredient Notification (NDIN) with clear safe intake limits (3 mg/day for long-term use, 5 mg/day for up to 90 days), making it the world’s first notified microalgae-derived fucoxanthin raw material. In the EU, *O. aurita* passed the substantial equivalence assessment by France in 2002 and was officially included in the EU Novel Food Catalog in 2020, approved for use in general foods and dietary supplements; the ethanol extract of *P. tricornutum* received a positive EFSA scientific opinion in 2023, confirming safety at proposed intake levels, and awaits formal adoption by the European Commission. Japan’s self-notification system for Foods with Function Claims (FFC) has widely accepted brown algae-derived fucoxanthin, with growing market acceptance for microalgae-derived ingredients. In Australia and New Zealand, fucoxanthin can be marketed as a complementary medicine via TGA notification, but general food use requires novel food assessment. Overall, regulatory hurdles remain a major bottleneck for market penetration of novel marine microalgae ingredients.

In the evaluation system of microalgae production processes, single intracellular content per dry weight cannot fully reflect the comprehensive efficiency of industrial production. Some extreme conditions can increase the intracellular pigment proportion but severely inhibit cell growth, resulting in lower actual yield per unit volume and no economic feasibility. Volumetric productivity (unit: mg/(L·d))—which integrates three dimensions of biomass concentration, intracellular pigment content and production cycle—is recognized as the core indicator for evaluating the industrial value of production processes in the field of algal biotechnology [11]. Using volumetric productivity as the core metric, this review systematically screens high-yield regulation studies of the two diatoms, summarizes enhancement effects of nutrition, light, inducers and strain improvement, and supplements intracellular content data. Combined with downstream processing, safety profiles and industrial progress, a comparative analysis is provided to support process optimization and industrial implementation of microalgae-derived fucoxanthin.

## 2. Efficient Regulation of Fucoxanthin Accumulation in *P. tricornutum*

### 2.1. Nitrogen Source Regulation

Nitrogen is a core nutrient factor for the synthesis of photosynthetic proteins and light-harvesting pigments in *P. tricornutum*, and directly determines cell growth rate and fucoxanthin biosynthesis level [12]. Although nitrogen deficiency stress can induce intracellular lipid accumulation, it leads to rapid degradation of light-harvesting complexes, synchronous decrease in fucoxanthin content and biomass, and significant reduction in final volumetric productivity. Therefore, sufficient and continuous nitrogen supply is a necessary prerequisite for high yield.

At the molecular level, nitrogen sufficiency upregulates the methylerythritol phosphate (MEP) pathway to supply isoprenoid precursors for carotenoid biosynthesis, and maintains high transcription of fucoxanthin-chlorophyll a/c binding protein (FCP) genes [13]. As fucoxanthin is predominantly bound to FCP complexes as a functional pigment, stabilization of these assemblies under nitrogen-replete conditions directly drives its sustained accumulation [14]. Nitrogen starvation, by contrast, triggers FCP degradation to recycle nitrogen, redirecting carbon flux toward storage lipids and chrysolaminarin rather than pigment biosynthesis [15].

In terms of nitrogen addition strategies, one-time high-dose addition easily causes substrate inhibition and osmotic stress, while fed-batch or two-stage regulation maintains nitrogen homeostasis and simultaneously boosts biomass and pigment. Gao et al. [16] adopted a two-stage nitrogen regulation strategy: sufficient nitrogen supply in the logarithmic growth phase to promote growth, and maintained high nitrogen level in the stationary phase to induce pigment accumulation. A fucoxanthin volumetric productivity of 4.73 mg/(L·d) was achieved in a flat-plate photobioreactor, which was 30% higher than that under constant low nitrogen culture. Under mixotrophic culture with glycerol as organic carbon source, Yang et al. [12] applied a two-stage light-nitrogen regulation strategy with tryptone supplementation in the second stage, achieving a volumetric productivity of 8.22 mg/(L·d), which was 73.78% higher than that of one-stage single-dose culture.

For concentration optimization, Afonso et al. [17] reported that increasing sodium nitrate to 10× the basal f/2 level (8.82 mmol/L) yielded a maximum volumetric productivity of 3.37 mg/(L·d). Elshobary et al. [18] constructed a mixotrophic system via N/P ratio optimization, silicon removal and glycerol addition, combined with low-intensity blue light. Volumetric productivity increased 5-fold relative to the standard f/2 autotrophic control, and intracellular fucoxanthin content increased to 20.44 mg/g DW. Regarding nitrogen source types, Urea as the sole nitrogen source markedly boosted fucoxanthin production, achieving 2.35–3.36-fold higher yields than those obtained with ammonia, nitrate, or mixed nitrogen sources [19].

### 2.2. Light Regulation

As a light-harvesting pigment, fucoxanthin biosynthesis is co-regulated by light intensity, light quality and photoperiod. *P. tricornutum* is more sensitive to short-wavelength light: blue light exerts a significantly stronger inductive effect on fucoxanthin synthesis than long-wavelength red light, and green light also has a certain induction effect, which is consistent with its evolutionary adaptation to benthic low-light environments [12,20]. The strong blue-light induction is attributed to the specific blue-light photoreceptor aureochrome, which directly upregulates transcription of key carotenogenic genes (e.g., *psy*, *pds*, *crtiso5*) upon blue light absorption [21]. This mechanism is consistent with the ecological niche of pennate diatoms, where short-wavelength scattered light dominates [22].

However, although pure blue light can increase intracellular pigment proportion, it significantly inhibits biomass growth and does not yield the optimal volumetric productivity. To balance biomass and pigment content, two-stage light quality shift strategies have been widely applied: red-dominant light in the first stage (0–6 days) to promote rapid cell proliferation, and blue–green mixed light (60 μmol/(m^2^·s)) in the second stage to induce fucoxanthin synthesis, which can increase fucoxanthin yield by 68% [20]. Yang et al. [12] verified this strategy under mixotrophic culture: by shifting light quality from red:blue = 6:1 to 5:1 (enhanced blue light proportion) combined with tryptone addition, the fucoxanthin biomass concentration reached 6.52 g/L with a volumetric productivity of 8.22 mg/(L·d). Compared with one-stage constant light quality culture, biomass concentration and intracellular fucoxanthin content increased by 8% and 12%, respectively, which is the highest reported volumetric productivity record for *P. tricornutum* to date.

Additionally, alginate-derived oligosaccharides (AOS) promote biomass growth and fucoxanthin synthesis, and alleviate high-light inhibition. Che et al. [23] showed that under 50 μmol/(m^2^·s) illumination with optimal AOS concentration, the maximum fucoxanthin content per dry weight reached 19.70 mg/g after 16 days of culture, 34% higher than the control group. Even under 300 μmol/(m^2^·s) high light stress, AOS-treated biomass was nearly double that of the control, with 17% higher fucoxanthin content.

Photoperiod (light-dark, LD) is a key regulator of fucoxanthin accumulation in *P. tricornutum*. High-frequency intermittent light markedly elevates intracellular pigment content by transcriptionally activating carotenoid and light-harvesting complex (LHC) genes, while extreme photoperiods reduce volumetric yield through two distinct pathways. Continuous illumination triggers photoinhibition, and severely light-limited cycles restrict carbon fixation. Zheng et al. [24] identified LD 2:2 as the optimal regimen, which reached a peak volumetric pigment concentration of 2.02 μg/L at the stationary phase. Exponential-phase cellular fucoxanthin under this condition significantly exceeded levels under conventional LD 16:8 and continuous light (LD 24:0), and even the suboptimal LD 4:2 regimen increased pigment content by 28.6% relative to LD 16:8. This enhancement stems from coordinated upregulation of the carotenoid biosynthetic pathway that redirects carbon flux toward pigment synthesis. In contrast, continuous light induces photosystem II photodamage, and short light-limited cycles including LD 8:16 and LD 2:4 limit total carbon assimilation, both of which depress overall volumetric productivity.

### 2.3. Other Environmental Condition Regulation

Temperature also acts as a major driving factor for fucoxanthin biosynthesis in *P. tricornutum*, and pigment accumulation often shows an opposite trend to biomass formation. Zang et al. [25] reported that intracellular fucoxanthin content reached 12.8 mg/g dry weight at 24–25 °C, approximately 22% higher than at 20 °C, while cell growth was notably repressed under this condition. Fan et al. [26] further verified this temperature-dependent regulation pattern and demonstrated that temperatures above 28–30 °C cause severe disruption of carotenoid metabolism. At 30 °C, fucoxanthin content declined significantly compared with the optimal temperature group, coupled with pronounced downregulation of key biosynthetic genes *psy*, *pds*, *lcyb* and *zds*; their study also revealed that exogenous cis-zeatin could mitigate high-temperature stress and restore fucoxanthin biosynthesis by upregulating these carotenogenic genes. For volumetric productivity, 18–22 °C represents the optimal trade-off range balancing cell proliferation and pigment synthesis, and this decoupling characteristic provides a theoretical basis for the two-stage cultivation strategy in practical production.

Salinity modulates fucoxanthin volumetric productivity in a unimodal pattern. Moderately high salinity induces intracellular pigment enrichment as an antioxidant defense against osmotic stress, which partially offsets biomass loss and maintains stable yield across a wide salinity window. Using a urea-amended palm oil mill effluent (POME) culture system and the Box–Behnken response surface methodology, Nur et al. [27] determined the optimal salinity range to be 25–31 practical salinity units (psu). Extreme hyposalinity at 5 psu reduced volumetric productivity to one-third of the peak value observed at 35 psu, while above the 31 psu threshold, cell density declined slightly but productivity remained stable up to 35 psu. Outdoor raceway trials with F/2 medium further validated this trend. Cellular fucoxanthin peaked at 18.65 mg/g DW at 20 psu, and no significant difference was detected across the 20–30 psu range despite a minor reduction in biomass at higher salinity levels [28]. Accordingly, a salinity range of 25–31 psu is recommended for industrial production to ensure both robust cell growth and efficient pigment synthesis [29].

### 2.4. Enhancement by Exogenous Inducers

Phytohormone inducers can further improve fucoxanthin productivity by regulating metabolic pathways, which is a low-cost enhancement method without changing the basic culture process. For single phytohormone addition, Dong et al. [30] applied 2.5 mg/L methyl jasmonate under low temperature (12 °C) and high light (130 μmol m^−2^ s^−1^) in a two-stage strategy, increasing productivity by 152.36% relative to control.

For hormone combinations, Deng et al. [31] found that indole-3-acetic acid (0.37 mg/L) combined with salicylic acid (0.4 mg/L) was the optimal combination, achieving a fucoxanthin productivity of 4.53 mg/(L·d). After further compounding with 10 mg/L calcium lignosulfonate, scale-up verification in a 5 L photobioreactor yielded a fucoxanthin content of 16.61 mg/g and volumetric productivity of 6.93 mg/(L·d). Compared with the non-supplemented group, biomass productivity, fucoxanthin content and productivity increased significantly by 28.42%, 40.05% and 65.00%, respectively. Furthermore, synergistic optimization of nutrients and light environment can also significantly increase fucoxanthin content. Elshobary et al. [18] optimized nitrogen-phosphorus ratio, removed silicon, added glycerol (2 g/L), and cultured under low-intensity blue light (100 μmol/(m^2^·s)), achieving a biomass of 1.6 g/L and fucoxanthin content of 20.44 mg/g, providing important reference for photo-fermentation processes.

### 2.5. Strain Improvement via Breeding and Metabolic Engineering

In addition to culture process optimization, mutation breeding and metabolic engineering are core technical routes to break through the fucoxanthin synthesis bottleneck of wild strains. For mutation breeding, Liu et al. [32] obtained mutant populations with significantly improved fucoxanthin yield and genetic stability via atmospheric and room temperature plasma (ARTP) mutagenesis combined with adaptive evolution screening. In a 10,000 L large-scale photobioreactor, the mutant population achieved a biomass of 0.59 g/L (18% higher than wild type), fucoxanthin concentration of 7.29 mg/L (32.79% higher) and volumetric productivity of 1.47 mg/(L·d) (54.74% higher). This study verified the scale-up feasibility of mutation breeding in a 10 m^3^ reactor, representing a successful case of translating mutant strains from laboratory to pilot scale.

For metabolic engineering, the mature genetic transformation system of *P. tricornutum* enables precise modification. Cao et al. [33] identified the carotenoid isomerase family protein CRTISO5 as fucoxanthin synthase; knockout mutants completely lost fucoxanthin, confirming that CRTISO5 is a key enzyme in the fucoxanthin biosynthetic pathway. On this basis, Truong et al. [14] systematically investigated the correlation between fucoxanthin biosynthesis and fucoxanthin-chlorophyll a/c binding protein (FCP) complexes under different light intensities. They found that FCP antenna complexes were significantly enriched under low light, with fucoxanthin content and volumetric productivity reaching 1.7 mg/g DW and 2.12 mg/(L·d), respectively, while only 0.54 mg/g DW and 0.79 mg/(L·d) under high light. Cen et al. [34] simultaneously overexpressed two key carotenogenic genes, 1-deoxy-D-xylulose-5-phosphate synthase and lycopene cyclase, achieving a fucoxanthin content of 6.53 mg/g DW without affecting normal physiological characteristics and biomass accumulation. In addition, Hao et al. [35] overexpressed *CMK* and *CMS* genes with low transcription levels in the MEP pathway; the overexpression lines showed significantly enhanced growth and photosynthetic efficiency, with fucoxanthin content increased by 1.83 and 1.82 times, respectively.

### 2.6. Enhanced Fucoxanthin Production Using Organic Wastewater as Substrate

Coupling microalgae-based wastewater treatment with high-value product biosynthesis is a core strategy to realize nutrient valorization of waste streams within the bio-circular economy framework. *P*. *tricornutum* cultivation has been successfully applied to livestock and food industrial wastewater systems to achieve the dual target of high-value production and pollution reduction.

Jiang et al. [36] adopted filtration-pretreated swine farm wastewater as the cultivation substrate, adjusted the volume ratio of wastewater to clean water, and supplemented the salinity to 20 psu for photoautotrophic culture in photobioreactors. The optimal comprehensive performance was achieved at a volume ratio of 1:1. After 20 days of cultivation under this condition, the biomass concentration reached 1.54 ± 0.40 g/L with the highest nutrient removal efficiency, and the volumetric fucoxanthin yield reached 28.41 ± 2.41 mg/L, which was comparable to the yield of standard f/2 medium.

Russo et al. [37] employed three food industrial wastewater by-products, i.e., cheese whey, beet molasses and corn steep liquor, as alternative nutrient substrates to realize highly efficient enhanced fucoxanthin production in *P. tricornutum*. The medium formulation was optimized through response surface methodology combined with central composite design, and the optimal wastewater-based medium composition was finally determined as 33 mL/L hydrolyzed cheese whey, 2.3 g/L beet molasses and 2.24 g/L corn steep liquor. Under this condition, the volumetric fucoxanthin yield of *P. tricornutum* reached 3.64 mg/L, and the fucoxanthin content per unit dry cell weight reached 1.79 mg/g, which was approximately 2.7 times the level obtained with standard BG11 medium.

### 2.7. Large-Scale Cultivation

For large-scale cultivation, raceway ponds, flat-plate photobioreactors and column photobioreactors can all support stable culture of *P. tricornutum*. Song et al. [28] carried out large-scale cultivation in outdoor raceway ponds with systematic optimization of salinity, pH and nitrogen source. Under optimal conditions (salinity 20 psu, pH 8.0), the maximum cell density reached 5.8 × 10^6^ cells/mL with fucoxanthin content of 17.79 mg/g. For nitrogen source selection, ammonium bicarbonate was more favorable for fucoxanthin accumulation (content 9.96 mg/g), verifying the feasibility of producing fucoxanthin via outdoor open raceway pond cultivation of *P. tricornutum*. Closed photobioreactors enable more precise environmental regulation, and pigment accumulation stability is significantly better than open systems. Aslanbay Guler et al. [38] scaled up *P. tricornutum* stepwise from 1000 mL flasks to 2 L and 7 L flat-plate photobioreactors, and fucoxanthin yield increased to 2.3 and 2.6 times that of flasks (1.05 mg/g dry weight in flasks), verifying the feasibility of flat-plate photobioreactors in scale-up culture.

Despite promising laboratory results, industrial scale-up faces inherent engineering challenges. As reactor volume increases, severe light attenuation causes uneven distribution, reduces average light utilization, and typically lowers volumetric productivity by 30–40% relative to optimized flask conditions [39]. Aeration and mixing shear stress can also cause cell damage and pigment degradation [40]. Systematic studies on light distribution optimization, mass transfer enhancement and shear control in large-scale fucoxanthin production remain scarce, representing a major gap between laboratory findings and industrial deployment.

### 2.8. Summary of Studies on P. tricornutum

The above sections have systematically reviewed high-yield regulation strategies for fucoxanthin accumulation in *P. tricornutum* across nutrient manipulation, environmental optimization, exogenous inducer application, and strain improvement. To facilitate intuitive cross-comparison of production efficiency among different processes, the core parameters and yield indicators of representative studies are summarized in Table 1.

## 3. Efficient Regulation of Fucoxanthin Accumulation in *Odontella aurita*

### 3.1. Nitrogen Source Regulation

Nitrogen supply level is the core regulatory factor for fucoxanthin accumulation in *O. aurita*. Nitrogen deficiency can induce chrysolaminarin accumulation as the main storage substance, but leads to degradation of light-harvesting complexes and rapid pigment decomposition; continuous nitrogen supplementation maintains the active state of the synthesis pathway and simultaneously increases biomass and pigment content. Xia et al. [41] reported that under low nitrate supply, fucoxanthin volumetric concentration and productivity were 27.11 mg/L and 2.71 mg/(L·d), respectively, which increased to 76.73 mg/L and 7.67 mg/(L·d) under sufficient nitrate conditions. On this basis, Xia et al. [42] further compared three nitrogen supply modes: initial low nitrogen (6 mmol/L), initial high nitrogen (18 mmol/L) and high nitrogen + fed-batch supplementation. The results showed that the fed-batch group achieved the highest fucoxanthin volumetric productivity of 6.01 mg/(L·d), which was 4.32 times that of the low nitrogen group and 1.42 times that of the high nitrogen group. For large-scale cultivation, Xia et al. [41] carried out culture in a bubble column photobioreactor. Under low light intensity and sufficient nitrogen conditions, the maximum biomass concentration of *O. aurita* reached 4.24 g/L, fucoxanthin content ranged from 18.01 to 21.67 mg/g dry weight, with a corresponding volumetric productivity of 7.67 mg/(L·d).

### 3.2. Light Regulation

*O. aurita* is a typical red-light-preferring diatom, and red light promotes its growth and fucoxanthin accumulation more effectively than blue light and white light [41,43]. The red-light preference is associated with its centric diatom origin and upper-water ecological niche [44]. Centric diatoms generally encode more red/far-red photoreceptors (e.g., phytochrome-like proteins) than pennate diatoms, and red light matches the spectral composition of surface seawater, supporting higher photosynthetic efficiency and faster biomass accumulation [45]. Zhang et al. [43] cultured *O. aurita* in an auto-controlled photobioreactor. When red and blue light were mixed at a ratio of 8:2, both biomass productivity and fucoxanthin productivity reached peaks: 570 mg/(L·d) and 9.41 mg/(L·d), respectively. This is the highest reported photoautotrophic productivity for *O. aurita*, with pilot-scale verification confirming its commercial feasibility.

### 3.3. Other Environmental Condition Regulations

Temperature, salinity and silicate also have significant effects on fucoxanthin productivity of *O. aurita*. An et al. [46] systematically analyzed environmental factors for the *O. aurita* strain OAOSH22 isolated from the east coast of Korea, and determined its optimal temperature as 21 °C, optimal salinity as 0.5 M NaCl (~29.2 psu), and optimal light intensity as 88 μmol m^−2^ s^−1^. Under these conditions, its fucoxanthin content reached 7.1 mg/g dry weight. As a diatom, *O. aurita* has special requirements for silicate for growth. Using high-silicon Jeju lava seawater to prepare medium increased growth by 1.8-fold; further medium optimization increased biomass by 15.6 times and fucoxanthin yield by 7.5 times [46]. This low-cost medium formulation provides a practical solution for production cost reduction in industrial scale-up.

### 3.4. Summary of Studies on O. aurita

Current research on fucoxanthin biosynthesis regulation in *O. aurita* mainly focuses on nitrogen supply regimes and light condition optimization. The production performance of typical regulation schemes is compiled in Table 2, providing a quantitative reference for subsequent process development.

## 4. Extraction and Purification Technologies

### 4.1. Green Extraction Technologies for Crude Fucoxanthin

Downstream processing is a critical cost-determining step in industrial fucoxanthin production, and green, efficient extraction technologies remain core research priorities in the marine bioproduct engineering. Organic solvent extraction is the most widely applied baseline approach. Kim et al. [47] screened five solvents (acetone, ethanol, water, n-hexane, ethyl acetate) for fucoxanthin extraction from *P. tricornutum*, and identified ethanol as the optimal candidate with a maximum yield of 15.71 mg/g lyophilized biomass, significantly outperforming n-hexane, the traditional solvent used in commercial production.

Supercritical CO_2_ extraction, a benchmark green technology for marine bioactives, features negligible solvent residue, minimal thermal degradation, and high retention of pigment bioactivity. Ruiz-Domínguez et al. [48] optimized the process parameters for supercritical CO_2_ extraction of fucoxanthin from *P. tricornutum* by evaluating temperature, pressure, and ethanol co-solvent concentration. The highest crude extract yield (24.41% of dry weight) was achieved at 30 MPa and 30 °C with 30% (v/v) ethanol as co-solvent; increasing ethanol to 40% under identical pressure and temperature raised fucoxanthin content in the crude extract to 85.03 mg/g, with a corresponding recovery of 66.60%. Other emerging green technologies, including pressurized liquid extraction (PLE) and microwave-assisted extraction (MAE), markedly reduce extraction time and solvent usage. Gilbert-López et al. [49] compared the two approaches for fucoxanthin recovery from diatom biomass, and confirmed that PLE achieved higher yields under optimized temperature and pressure, making it more suitable for scaled-up batch production.

### 4.2. Purification Technologies for High-Purity Fucoxanthin

Following crude extraction, further purification is required to obtain high-purity fucoxanthin suitable for food and pharmaceutical uses. Column chromatography remains the predominant technique for this purpose. Zhu et al. [50] developed a gradient elution protocol using ODS open-column chromatography: initial elution with 70% (v/v) ethanol removed most of polar impurities, and subsequent elution with 80% (v/v) ethanol yielded fucoxanthin-enriched fractions with an overall recovery of (62.07 ± 2.68)%. To further improve product purity, Zhao et al. [51] proposed a two-step strategy combining silica gel column separation and hydrophilic-lipophilic balance (HLB) solid-phase extraction, which effectively eliminated non-polar lipids and residual impurities. The final product reached 92.03% purity with 76.67% recovery, satisfying quality criteria for high-end functional food ingredients.

For integrated biorefinery applications, high-concentration ethanol enables co-recovery of multiple high-value components: Delbrut et al. [52] reported that 96% (v/v) ethanol achieved simultaneous extraction of 95% fucoxanthin and 89% eicosapentaenoic acid (EPA) within 24 h, supporting cost reduction through multi-product synergy.

### 4.3. Stability Bottleneck and Formulation Strategies

Despite extraction advances, fucoxanthin’s chemical instability remains a critical industrial bottleneck [53]. It is highly susceptible to oxidative degradation and isomerization under light, heat and acidic conditions, leading to rapid bioactivity loss during processing, storage and gastrointestinal digestion. Formulation strategies including spray-dried microencapsulation, oil-in-water nanoemulsions and cyclodextrin inclusion complexes have been developed to improve storage stability, water solubility and oral bioavailability [54,55]. Among them, spray-dried microcapsules retain over 80% of fucoxanthin activity after 3 months of room-temperature storage, representing the most technically mature approach for industrial scale-up [56].

## 5. Systematic Comparison of Fucoxanthin Production Performance Between the Two Diatoms

### 5.1. Comparison of Productivity Indicators

In volumetric productivity, *O. aurita* holds an overall advantage: the laboratory-optimized maximum reaches 9.41 mg/(L·d) [43], compared with 8.22 mg/(L·d) for *P. tricornutum* [12]. The core advantage of *O. aurita* stems from stronger biomass accumulation: it reaches up to 6.36 g/L in reactor culture [41], significantly higher than the 3–4 g/L typical of *P. tricornutum*. Even with slightly lower intracellular pigment proportion, the total yield per unit volume is still higher. In intracellular content, the two strains are comparable: *P. tricornutum* reaches 20.44 mg/g DW under glycerol mixotrophy [18], while *O. aurita* reaches 23.30 mg/g DW under optimized nitrogen supply [42]. Both fall in the 18–24 mg/g range under optimized conditions.

From a cost-structure perspective, the economics differ by product purity grade. For low-purity crude extracts, brown macroalgae extraction remains lower-cost, with raw material procurement and purification losses accounting for over 70% of total cost [57,58]. For high-purity (HPLC ≥ 90%) fucoxanthin, however, diatom-based production has emerged as cost-competitive: purified diatom fucoxanthin costs approximately 36,600 USD/kg [58], compared with over 50,000 USD/kg from brown macroalgae [59]. This advantage stems from higher pigment content, fewer purification steps and lower purification losses.

From a techno-economic perspective, the high biomass characteristic of *O. aurita* endows it with stronger cost-sharing ability in large-scale cultivation. Meanwhile, this strain can co-produce EPA and chrysolaminarin simultaneously, which can further reduce comprehensive production costs via multi-product synergy. *P. tricornutum*, with its mature genetic manipulation platform, has greater potential for leapfrog productivity improvement via metabolic engineering. Compared with the traditional brown macroalgae extraction route, the two diatoms have order-of-magnitude advantages in volumetric production efficiency.

### 5.2. Methodological Boundaries for Cross-Study Productivity Comparison

Direct cross-study comparison of absolute volumetric productivity values has inherent methodological limitations, which must be clarified to avoid overinterpretation of inter-strain numerical differences. Volumetric productivity, while serving as the most intuitive indicator of production efficiency, generally lacks direct comparability across different studies, primarily due to the non-linear characteristics of cultivation modes and scale-up processes [60]. Khaw et al. [61] note that existing literature predominantly focuses on reporting the peak content of fucoxanthin, and volumetric productivity calculated based on a single day of peak yield is insufficient to reflect the actual production performance over the entire cultivation cycle.

Furthermore, there is no linear correlation between reactor scale and fucoxanthin yield, and the uniformity of light field distribution is also a core engineering factor governing the level of volumetric productivity. For instance, scale-up experiments on *O. aurita* by Xia et al. confirmed that when the light path of the photobioreactor increased from 3 cm to 6 cm and the working volume expanded from 75 L to 150 L, biomass concentration and fucoxanthin volumetric productivity decreased by 39.4% and 39.3%, respectively. In contrast, under comparable light path conditions, when the cultivation system was scaled up from a 320 mL bench-scale bubble column reactor to a 75 L flat-panel photobioreactor, volumetric productivity only declined from 7.96 mg/(L·d) to 6.92 mg/(L·d) [41]. These findings indicate that direct cross-study comparison of volumetric productivity values, without accounting for core prerequisites such as reactor configuration, light path, incident light intensity, and cultivation strategies, lacks rigorous methodological validity. Future studies are required to establish standardized specifications for process parameters and a unified performance evaluation framework to enhance the comparability of results across different investigations.

### 5.3. Comparison of Environmental Response Patterns

For nitrogen nutrition, both strains follow the common rule that sufficient nitrogen promotes fucoxanthin accumulation while nitrogen deficiency causes rapid pigment degradation; consequently, phased or fed-batch nitrogen supplementation is recognized as a core strategy for sustaining high productivity in both strains. In *O. aurita*, fucoxanthin behaves as a primary light-harvesting, nitrate-replete conditions concurrently elevate biomass and pigment content; in *P. tricornutum*, fucoxanthin occupies a light harvesting and photoprotection dual role, and staged organic nitrogen supplementation synergizes with light quality to push volumetric productivity to over 8 mg/(L·d) [12,41].

Marked species-specific light quality responses are observed. *P. tricornutum* is highly sensitive to short-wavelength blue light, which strongly induces fucoxanthin biosynthesis, a red to blue–green shift strategy (red in phase 1 for biomass, blue:green in phase 2 for pigment) raised the volumetric yield to 12.18 mg/L under 60 μmol/(m^2^·s). *O. aurita*, by contrast, is a red-light-preferring strain, with mixed red:blue at 8:2 (300 μmol/(m^2^·s), internally illuminated PBR) delivering the highest biomass (5.65 g/L) and fucoxanthin productivity (9.41 mg/(L·d)) reported for photoautotrophic culture. This divergence is rooted in differences in photoreceptor systems and ecological niche adaptation between pennate and centric diatoms, as detailed in previous sections.

Both strains share similar response patterns to temperature and salinity, with minor differences in optimal ranges. The optimal growth temperature for both is 16–24 °C; moderate warming (24–25 °C) elevates intracellular pigment content but suppresses biomass growth, while temperatures above 28 °C trigger widespread downregulation of carotenogenic genes and marked pigment degradation. Salinity induces a unimodal response in both strains, with peak volumetric productivity at 25–31 psu; moderately high salinity drives pigment enrichment via antioxidant defense, partially offsetting biomass loss. Notably, *O. aurita* has a higher silicate demand due to its thicker silicified frustule, making silicate availability an additional limiting factor for its large-scale cultivation.

Despite these differences, a two-stage light quality strategy—growth promotion in phase 1 and pigment induction in phase 2—is applicable to both strains, with only optimal parameter ratios differing [12,20,43]. Both strains are adapted to medium-low light, with light saturation points below 100 μmol/(m^2^·s); high light causes photooxidative damage and pigment degradation in both.

### 5.4. Techno-Economic Analysis and Cost Structure of Different Production Routes

Consistent with the core industrial bottleneck of high production costs, techno-economic analysis confirms that cost reduction through process optimization and multi-product co-production is the primary direction for future industrial scaling.

From a techno-economic standpoint, *O. aurita*’s high biomass endows it with stronger cost-sharing ability in large-scale cultivation. Its ability to co-produce EPA and chrysolaminarin further reduces comprehensive costs via multi-product synergy. *P. tricornutum*, with its mature genetic manipulation platform, has greater potential for step-change productivity improvement via metabolic engineering.

Compared with the traditional brown macroalgae extraction route, the two diatoms have order-of-magnitude advantages in volumetric production efficiency. Based on current process levels, about 5–9 g of fucoxanthin can be produced per cubic meter of algal culture per day, with a production cycle of only 10–16 days, free from seasonal climate restrictions and available for continuous production year-round. Notably, closed photobioreactor culture eliminates heavy metal and marine pollutant enrichment risks associated with wild macroalgae, which is a critical advantage for high-purity pharmaceutical and food-grade products.

The cost structure of fucoxanthin production differs markedly between brown macroalgae and microalgae-based systems, with fundamental distinctions in their core cost drivers and cost reduction potential. For production routes relying on brown macroalgae as feedstock, costs are predominantly concentrated in downstream extraction and purification processes. The natural fucoxanthin content in brown macroalgae generally ranges merely from 0.001% to 0.356% dry weight (DW), far lower than the level in microalgae [61]. Producing a unit mass of the target compound requires processing enormous volumes of raw material and implementing multiple separation and purification steps, which drives the extremely high proportion of extraction and purification costs in the total production expenditure. Furthermore, limited by the inherent physiological characteristics of macroalgal species, the fucoxanthin content in brown macroalgae can barely be elevated by orders of magnitude via artificial regulation, resulting in very restricted cost reduction space for the extraction link of this production pathway [42,62]. In sharp contrast, the production cost of microalgae-derived fucoxanthin is mainly incurred in the upstream cultivation stage, i.e., the large-scale acquisition of microalgal biomass. A techno-economic analysis of the co-production system with *P. tricornutum* cultivated in flat-panel airlift photobioreactors indicates that costs directly related to biomass production—including lighting energy consumption, medium components, equipment depreciation, and operation and maintenance expenses—account for more than 70% of the total production cost of fucoxanthin, while the downstream extraction and purification section contributes a relatively small share [63]. Unlike the cost reduction bottleneck of macroalgae constrained by natural attributes, the growth and carotenoid metabolism of microalgae are highly tunable. By optimizing multi-dimensional process conditions such as light parameters, nutrient supply strategies, cultivation modes, and reactor configurations, both biomass concentration and intracellular fucoxanthin accumulation can be synchronously improved. This in turn amortizes the fixed cost per unit product through enhanced volumetric productivity, granting the microalgae-based route significant potential for production cost reduction [29,61].

### 5.5. Industrial Potential and Commercial Depolyment Progress

In molecular biology and breeding potential, *P. tricornutum* has significant advantages. As a model diatom, its genome was sequenced early, and its genetic transformation system is mature. Technologies including CRISPR/Cas9, overexpression and gene knockout have all been established, with clear technical routes for further productivity improvement via precise metabolic engineering. Different degrees of productivity enhancement have been achieved via transcription factor overexpression and key enzyme modification [64,65]. In contrast, *O. aurita* has a weak foundation in molecular biology research, incomplete genomic information and an unestablished genetic transformation system, which is consistent with the overall early-stage research status of this strain. Further productivity improvement mainly relies on culture condition optimization and mutation breeding, with more prominent technical bottlenecks. However, with wider access to sequencing technology, its omics research is advancing, with considerable metabolic engineering potential.

From a safety perspective, both diatoms have substantial toxicological evidence supporting food use. *P. tricornutum* extract showed no observed adverse effect level above the proposed daily intake in 90-day subchronic studies, with no genotoxicity, skin sensitization or acute oral toxicity identified in standard evaluations [10]. *O. aurita* has a long history of safe use as an aquaculture feed ingredient in Europe, and its whole-cell biomass has been confirmed to be non-toxic in acute and repeated-dose toxicity tests [66].

In industrial implementation, both strains have moved beyond laboratory technical verification, and many enterprises worldwide have deployed large-scale production with different commercialization paths and market positioning. *P. tricornutum* is a relatively fast-advancing strain for commercial microalgae-derived fucoxanthin. The standardized extract FucoVital™ developed by Israel-based Algatechnologies is the world’s first commercially applied microalgae-derived fucoxanthin raw material. It has completed the FDA New Dietary Ingredient Notification (NDIN) in the US and a positive EFSA opinion in the EU. *O. aurita*’s commercial deployment is concentrated in the EU, with French and German enterprises operating closed large-scale cultivation, supplying whole-cell powder and standardized extracts for food, aquaculture feed and nutritional supplements. Its core commercial advantage lies in fucoxanthin-EPA-chrysolaminarin co-production, but its global regulatory coverage remains narrow, with major non-EU markets still in the application stage.

## 6. Conclusions and Perspectives

Different from previous mechanism-focused reviews, this work provides a full-chain, industry-oriented reference for fucoxanthin industrial production with volumetric productivity as the core evaluation criterion, directly addressing the key practical issues of cost control, process optimization, product stabilization and regulatory compliance in large-scale production. Both *P. tricornutum* and *O. aurita* are highly potential fucoxanthin-producing diatoms, and both achieve efficient fucoxanthin accumulation via continuous high nitrogen supply and medium-low light intensity culture. Taking volumetric productivity as the core evaluation standard, *O. aurita* exhibits strong potential for industrial translation, driven by its robust biomass accumulation capacity. Notably, under controlled laboratory conditions, its reported peak productivity reaches 9.41 mg/(L·d), suggesting promising economic viability, although comprehensive validation via pilot- and industrial-scale studies remains limited at this stage. *P. tricornutum* has comparable intracellular pigment content and more mature strain improvement technologies, suitable for further breaking through the production capacity ceiling via metabolic engineering, with a current optimal process productivity of 8.22 mg/(L·d). Both strains demonstrate markedly higher fucoxanthin production efficiencies than conventional extraction processes using brown macroalgae, establishing a robust technical foundation for their development as promising alternative or complementary sources of fucoxanthin.

From the perspective of industrial development, the two strains have attained critical regulatory milestones in the European Union and the United States, breaking through core market access barriers for specific application scenarios and laying the groundwork for commercialization. However, the acquisition of full market access authorization remains subject to multiple constraints, including product purity specifications, intended use, production processes, and regulatory requirements across different jurisdictions. With the advancement of China’s review and approval procedures for novel food ingredients, the market potential of microalgae-derived fucoxanthin in the domestic market is expected to be further unlocked. Nevertheless, the current global supply of high-purity fucoxanthin is still dominated by the brown algae extraction route, which faces challenges such as limited production capacity and notable quality fluctuations. Microalgae-derived products account for a relatively small share of the market, and high production costs remain the core bottleneck restricting their large-scale application. From the perspective of industrial development, both strains have achieved phased regulatory breakthroughs in the EU and US markets, removing part of the core obstacles for commercial application. With the advancement of new food raw material application in China in the future, the domestic market space for microalgae-derived fucoxanthin will be further released. However, the current global high-purity fucoxanthin supply is still dominated by brown algae extraction, facing multiple problems such as limited production capacity, large quality fluctuation and market conceptual confusion. The proportion of microalgae-derived products is still low, and high production cost is the core restricting factor.

Looking ahead, several key research directions are identified to accelerate industrial translation:

(a) Photobioreactor design and process intensification: Develop customized reactors and fed-batch processes matched to each strain’s light quality characteristics, optimize internal light distribution and mass transfer, address scale-up productivity attenuation, further improve volumetric productivity, and reduce energy and medium costs.
(b) Synthetic biology tool development and strain engineering: Combine mutation breeding and metabolic engineering to break through wild-type biosynthesis bottlenecks. In particular, genetic manipulation tools for *O. aurita* are urgently needed to unlock its high-biomass potential via key gene modification and transcription factor regulation.
(c) Optimization pathways for green production of fucoxanthin: There remains ample optimization potential for fucoxanthin production via the cultivation of *P. tricornutum* using organic wastewater. A coupled system integrating wastewater purification, flue gas carbon sequestration, and the co-production of fucoxanthin and eicosapentaenoic acid (EPA) can be established. Metabolic engineering and stress regulation can be applied to enhance the substrate tolerance of algal strains and boost pigment biosynthesis efficiency, paired with low-cost extraction and purification processes. In parallel, adapted large-scale cultivation technologies shall be developed, so as to provide a feasible paradigm for the green, low-cost production of fucoxanthin and the high-value utilization of organic wastewater.
(d) Safety evaluation and regulatory standardization: Establish unified quality standards and detection methods for microalgae-derived fucoxanthin, supplement complete toxicological data packages, and promote harmonization of global regulatory standards for marine microalgae food ingredients.
(e) Industrialization benefit analysis: In view of the ongoing disputes over economic viability and ambiguous environmental benefits in the industrialization of microalgae-derived fucoxanthin, it is necessary to construct a quantitative decision-making support system for the fucoxanthin industry based on the coupled approach of techno-economic analysis (TEA) and Life Cycle Assessment (LCA). The integrated application of these two analytical tools can clarify the commercial competitiveness threshold of microalgae-derived fucoxanthin, provide a scientific basis for process optimization, industrial policy support and cross-jurisdictional regulatory mutual recognition, and thus avoid the tendencies of blind optimism or excessive conservatism in the process of industrial development.

## Figures and Tables

**Table 1:** Summary of studies on high-yield fucoxanthin regulation in *Phaeodactylum tricornutum*.

Regulation Strategy	Culture System	Fucoxanthin Volumetric Productivity (mg/(L·d))	Content per Dry Weight (mg/g DW)	Reference
Two-stage light quality shift + nitrogen supplementation (mixotrophic)	Aerated flask	8.22	13.26	[12]
Phytohormones + calcium lignosulfonate induction	5 L photobioreactor	6.93	16.61	[31]
Two-stage nitrogen regulation	Flat-plate photobioreactor	4.73	—	[16]
High-concentration nitrate optimization	Aerated flask	3.37	—	[17]
Alginate-derived oligosaccharides (AOS) induction	Aerated flask	—	19.70	[23]
Nutrients + glycerol + low blue light optimization	Aerated flask	—	20.44	[18]
Two-stage light quality and intensity shift	Aerated flask	1.02	11.50	[20]
Outdoor raceway pond large-scale cultivation	Raceway pond	0.59	17.79	[28]

Note: Some studies took intracellular content as the core observation index without direct calculation of volumetric productivity, marked as “—” for auxiliary reference only.

**Table 2:** Summary of studies on high-yield fucoxanthin regulation in *O. aurita*.

Regulation Strategy	Culture System	Fucoxanthin Volumetric Productivity (mg/(L·d))	Content per Dry Weight (mg/g DW)	Reference
Red:blue light ratio = 8:2	20 L built-in LED PBR	9.41	—	[43]
18 mM nitrate + 100 μmol/(m^2^·s) low light	Bubble column photobioreactor	7.67	18.47	[41]
Initial high nitrogen + periodic fed-batch supplementation	Shake flask autotrophy	6.01	23.30	[42]

Note: Some studies took intracellular content as the core observation index without direct calculation of volumetric productivity, marked as “—“ for auxiliary reference only.

## Data Availability

No new data were created or analyzed in this study. Data sharing is not applicable to this article.

## References

[1] Yang S., Li J., Yan L., Wu Y., Zhang L., Li B., Tong H., Lin X. (2024). Molecular mechanisms of fucoxanthin in alleviating lipid deposition in metabolic associated fatty liver disease. J. Agric. Food Chem..

[2] Winarto J., Song D.G., Pan C.H. (2023). The role of fucoxanthin in non-alcoholic fatty liver disease. Int. J. Mol. Sci..

[3] Zhao D., Kwon S.-H., Chun Y.S., Gu M.-Y., Yang H.O. (2017). Anti-neuroinflammatory effects of fucoxanthin via inhibition of akt/nf-κb and mapks/ap-1 pathways and activation of pka/creb pathway in lipopolysaccharide-activated bv-2 microglial cells. Neurochem. Res..

[4] Din N.A.S., Mohd Alayudin A.S., Sofian-Seng N.-S., Rahman H.A., Mohd Razali N.S., Lim S.J., Wan Mustapha W.A. (2022). Brown algae as functional food source of fucoxanthin: A review. Foods.

[5] Noviendri D., Fithriani D., Hasrini R.F. (2021). Fucoxanthin, A Xanthophyll from Macro- and Microalgae: Extraction Techniques, Bioactivities and Their Potential Application in Nutra- and Cosmeceutical Industries. E3S Web Conf..

[6] Lourenço-Lopes C., Garcia-Oliveira P., Carpena M., Fraga-Corral M., Jimenez-Lopez C., Pereira A.G., Prieto M.A., Simal-Gandara J. (2020). Scientific approaches on extraction, purification and stability for the commercialization of fucoxanthin recovered from brown algae. Foods.

[7] Anjana K., Arunkumar K. (2024). Brown algae biomass for fucoxanthin, fucoidan and alginate; update review on structure, biosynthesis, biological activities and extraction valorisation. Int. J. Biol. Macromol..

[8] Wu Y., Chaumier T., Manirakiza E., Veluchamy A., Tirichine L. (2023). PhaeoEpiView: An epigenome browser of the newly assembled genome of the model diatom *Phaeodactylum tricornutum*. Sci. Rep..

[9] An S.M., Cho K., Kim E.S., Ki H., Choi G., Kang N.S. (2023). Description and characterization of the *Odontella aurita* OAOSH22, a marine diatom rich in eicosapentaenoic acid and fucoxanthin, isolated from Osan Harbor, Korea. Mar. Drugs.

[10] Turck D., Bohn T., Castenmiller J., De Henauw S., Hirsch-Ernst K.I., Maciuk A., Mangelsdorf I., McArdle H.J., EFSA Panel on Nutrition, Novel Foods and Food Allergens (2023). Safety of an ethanolic extract of the dried biomass of the microalga *Phaeodactylum tricornutum* as a novel food pursuant to Regulation (EU) 2015/2283. EFSA J..

[11] Bauer C., Schmitz C., Corrêa R.G., Herrera C., Ramlov F., Oliveira E.R.D., Pizzato A., Varela L., Cabral D., Yunes R. (2019). In vitro fucoxanthin production by the *Phaeodactylum tricornutum* diatom. Stud. Nat. Prod. Chem..

[12] Yang R., Wei D. (2020). Improving fucoxanthin production in mixotrophic culture of marine diatom *Phaeodactylum tricornutum* by LED light shift and nitrogen supplementation. Front. Bioeng. Biotechnol..

[13] Truong T.Q., Park Y.J., Winarto J., Huynh P.K., Moon J., Choi Y.B., Song D.G., Koo S.Y., Kim S.M. (2024). Understanding the impact of nitrogen availability: A limiting factor for enhancing fucoxanthin productivity in microalgae cultivation. Mar. Drugs.

[14] Truong T.Q., Park Y.J., Koo S.Y., Choi J.-H., Enkhbayar A., Song D.-G., Kim S.M. (2023). Interdependence of fucoxanthin biosynthesis and fucoxanthin-chlorophyll a/c binding proteins in *Phaeodactylum tricornutum* under different light intensities. J. Appl. Phycol..

[15] Alipanah L., Rohloff J., Winge P., Bones A.M., Brembu T. (2015). Whole-cell response to nitrogen deprivation in the diatom *Phaeodactylum tricornutum*. J. Exp. Bot..

[16] Gao B., Chen A., Zhang W., Li A., Zhang C. (2017). Co-production of lipids, eicosapentaenoic acid, fucoxanthin, and chrysolaminarin by *Phaeodactylum tricornutum* cultured in a flat-plate photobioreactor under varying nitrogen conditions. J. Ocean. Univ. China.

[17] Afonso C., Braganca A.R., Rebelo B.A., Serra T.S., Abranches R. (2022). Optimal nitrate supplementation in *Phaeodactylum tricornutum* culture medium increases biomass and fucoxanthin production. Foods.

[18] Elshobary M.E., Abo-Shanab W.A., Ende S.S., Alquraishi M., El-Shenody R.A. (2025). Optimizing *Phaeodactylum tricornutum* cultivation: Integrated strategies for enhancing biomass, lipid, and fucoxanthin production. Biotechnol. Biofuels.

[19] Wu Z., Qiu S., Abbew A.W., Chen Z., Liu Y., Zuo J., Ge S. (2022). Evaluation of nitrogen source, concentration and feeding mode for co-production of fucoxanthin and fatty acids in *Phaeodactylum tricornutum*. Algal Res..

[20] Song Q., Liu C., Xu R., Cai L. (2025). Enhancement of fucoxanthin accumulation in *Phaeodactylum tricornutum* by light quality and intensity shift strategy. Chem. Eng. J..

[21] Im S.H., Lepetit B., Mosesso N., Shrestha S., Weiss L., Nymark M., Roellig R., Wilhelm C., Isono E., Kroth P.G. (2024). Identification of promoter targets by aureochrome 1a in the diatom *Phaeodactylum tricornutum*. J. Exp. Bot..

[22] Coesel S.N. (2024). More than a photoreceptor: Aureochromes are intrinsic to the diatom light-regulated transcriptional network. J. Exp. Bot..

[23] Che X., Fan D., Ding R., Wu H., Li Y. (2025). Alginate-derived oligosaccharides (AOS) enhance the biomass of *Phaeodactylum tricornutum* and fucoxanthin yield. J. Appl. Phycol..

[24] Zheng J., Yuan J., Wang X., Zhang D., Lin L., Shi N., Feng Y. (2026). Intermittent light enhances pigment production in the diatom *Phaeodactylum tricornutum*: A combined physiological and transcriptomic approach. Microbiol. Spectrum.

[25] Zang Z., Xie X., Zhao P., Huan L., Huang A., Zhang B., Pan G., Wang G. (2015). Effects of temperature and light on the growth and fucoxanthin content of *Phaeodactylum tricornutum*. Mar. Sci..

[26] Fan S., Li Y., Wang Q., Jin M., Yu M., Zhao H., Zhou C., Xu J., Li B., Li X. (2024). The role of cis-zeatin in enhancing high-temperature resistance and fucoxanthin biosynthesis in *Phaeodactylum tricornutum*. Appl. Environ. Microbiol..

[27] Nur M.M.A., Muizelaar W., Boelen P., Buma A.G.J. (2019). Environmental and nutrient conditions influence fucoxanthin productivity of the marine diatom Phaeodactylum tricornutum grown on palm oil mill effluent. J. Appl. Phycol..

[28] Song P., Liu L., Wei D. (2018). Scaling-up cultivation of *Phaeodactylum tricornutum* in open raceway pond and optimization of the culture conditions for fucoxanthin accumulation. Mod. Food Sci. Technol..

[29] Wang S., Wu S., Yang G., Pan K., Wang L., Hu Z. (2021). A review on the progress, challenges and prospects in commercializing microalgal fucoxanthin. Biotechnol. Adv..

[30] Dong X., Zhao M.H., Li C.X., Lin X., Ma N., Qiao H.J., Han D.F., Xu Y.J. (2025). Enhanced production of eicosapentaenoic acid and fucoxanthin in *Phaeodactylum tricornutum* via a two-stage strategy with multifactorial synergistic effects. J. Appl. Phycol..

[31] Deng J., Yang Z., Wei D. (2025). Combination of plant hormones promotes high-yield fucoxanthin production in mixotrophic *Phaeodactylum tricornutum*. Mod. Food Sci. Technol..

[32] Liu W., Liu J., Zou S., Long J., Zhang Q., He Y., Chen H., Liu Y., Fan Y., Hu Q. (2025). Optimizing fucoxanthin production in the diatom *Phaeodactylum tricornutum* using ARTP-evolved mutant consortia and a multifaceted approach in LED-driven photobioreactors. Algae.

[33] Cao T., Bai Y., Buschbeck P., Tan Q., Cantrell M.B., Chen Y., Jiang Y., Liu R.-Z., Ries N.K., Shi X. (2023). An unexpected hydratase synthesizes the green light-absorbing pigment fucoxanthin. Plant Cell.

[34] Cen S.-Y., Li D.-W., Huang X.-L., Huang D., Balamurugan S., Liu W.-J., Zheng J.-W., Yang W.-D., Li H.-Y. (2022). Crucial carotenogenic genes elevate hyperaccumulation of both fucoxanthin and β-carotene in *Phaeodactylum tricornutum*. Algal Res..

[35] Hao T.-B., Lu Y., Zhang Z.-H., Liu S.-F., Wang X., Yang W.-D., Balamurugan S., Li H.-Y. (2021). Hyperaccumulation of fucoxanthin by enhancing methylerythritol phosphate pathway in *Phaeodactylum tricornutum*. Appl. Microbiol. Biotechnol..

[36] Jiang J., Huang J., Zhang H., Zhang Z., Du Y., Cheng Z., Feng B., Yao T., Zhang A., Zhao Z. (2022). Potential integration of wastewater treatment and natural pigment production by *Phaeodactylum tricornutum*: Microalgal growth, nutrient removal, and fucoxanthin accumulation. J. Appl. Phycol..

[37] Russo G., Langellotti A., Verardo V., Martín-García B., Oliviero M., Baselice M., Di Pierro P., Sorrentino A., Viscardi S., Marileo L. (2023). Bioconversion of Cheese Whey and Food By-Products by *Phaeodactylum tricornutum* into Fucoxanthin and n-3 Lc-PUFA through a Biorefinery Approach. Mar. Drugs.

[38] Aslanbay Guler B., Deniz I., Demirel Z., Oncel S.S., Imamoglu E. (2019). Transition from start-up to scale-up for fucoxanthin production in flat plate photobioreactor. J. Appl. Phycol..

[39] Chen C.Y., Liu P.Y., Chang Y.H., Nagarajan D., Latagan M.J.D., Luna M.D.G.D., Chen J.H., Chang J.S. (2024). Optimizing cultivation strategies and scaling up for fucoxanthin production using *Pavlova* sp.. Bioresour. Technol..

[40] Aslanbay Guler B., Deniz I., Demirel Z., Oncel S.S., Imamoglu E. (2019). Comparison of different photobioreactor configurations and empirical computational fluid dynamics simulation for fucoxanthin production. Algal Res..

[41] Xia S., Wang K., Wan L., Li A., Hu Q., Zhang C. (2013). Production, characterization, and antioxidant activity of fucoxanthin from the marine diatom *Odontella aurita*. Mar. Drugs.

[42] Xia S., Gao B., Fu J., Xiong J., Zhang C. (2018). Production of fucoxanthin, chrysolaminarin, and eicosapentaenoic acid by *Odontella aurita* under different nitrogen supply regimes. J. Biosci. Bioeng..

[43] Zhang H., Gong P., Cai Q., Zhang C., Gao B. (2022). Maximizing fucoxanthin production in *Odontella aurita* by optimizing the ratio of red and blue light-emitting diodes in an auto-controlled internally illuminated photobioreactor. Bioresour. Technol..

[44] Sansone C., Russo M.T., Paris D., Pistelli L., Maselli M., Corato F., Tramice A., Iodice A., Margiotta F., Smerilli A. (2025). The color within light is a crucial cue that impacts key metabolites and vitamins in the diatom *Odontella aurita*. Front. Physiol..

[45] Volpe C., Büchel C. (2024). Function, Structure and Organization of Light-Harvesting Proteins in Diatoms. Diatom Photosynthesis: From Primary Production to High-Value Molecules.

[46] An S.M., Cho K., Kang N.S., Kim E.S., Ki H., Choi G., An H.S., Go G.M. (2024). Development of a cost-effective medium suitable for the growth and fucoxanthin production of the microalgae *Odontella aurita* using jeju lava seawater and agricultural fertilizers. Biomass Bioenerg..

[47] Kim S.M., Jung Y.-J., Kwon O.-N., Cha K.H., Um B.-H., Chung D., Pan C.-H. (2012). A potential commercial source of fucoxanthin extracted from the microalga *Phaeodactylum tricornutum*. Appl. Biochem. Biotechnol..

[48] Ruiz-Domínguez M.C., Salinas F., Medina E., Rincón B., Martín M.Á., Gutiérrez M.C., Cerezal-Mezquita P. (2022). Supercritical fluid extraction of fucoxanthin from the diatom *Phaeodactylum tricornutum* and biogas production through anaerobic digestion. Mar. Drugs.

[49] Gilbert-López B., Barranco A., Herrero M., Cifuentes A., Ibáñez E. (2017). Development of new green processes for the recovery of bioactives from *Phaeodactylum tricornutum*. Food Res. Int..

[50] Zhu J., Ye Y., Zhou C., Zhang J. (2024). Study on the separation and purification process of fucoxanthin from *Phaeodactylum tricornutum*. Chin. J. Mar. Drugs.

[51] Zhao X., Gao L., Zhao X. (2022). Rapid purification of fucoxanthin from *Phaeodactylum tricornutum*. Molecules.

[52] Delbrut A., Albina P., Lapierre T., Pradelles R., Dubreucq E. (2018). Fucoxanthin and polyunsaturated fatty acids co-extraction by a green process. Molecules.

[53] Gomez-Zavaglia A., Barros L., Prieto M.A., Cassani L. (2023). Recent progress in understanding the impact of food processing and storage on the structure–activity relationship of fucoxanthin. Foods.

[54] Sun X., Xu Y., Zhao L., Yan H., Wang S., Wang D. (2018). The stability and bioaccessibility of fucoxanthin in spray-dried microcapsules based on various biopolymers. RSC Adv..

[55] Wang S., Guo M., Jin Z. (2025). Innovative controlled-release systems for fucoxanthin: Research progress and applications. Pharmaceutics.

[56] Li H., Shang W., Wu S., Tan M., Wang H. (2024). Development of intestine-targeted microcapsules for enhanced delivery of fucoxanthin: A strategy to mitigate lipid accumulation in vitro and in vivo. Food Biosci..

[57] Pang Y., Duan L., Song B., Cui Y., Liu X., Wang T.J.B. (2024). A review of fucoxanthin biomanufacturing from *Phaeodactylum tricornutum*. Bioprocess Biosyst. Eng..

[58] Budiarso F.S., Leong Y.K., Chang J.-J., Chen C.-Y., Chen J.-H., Yen H.-W., Chang J.-S. (2025). Current advances in microalgae-based fucoxanthin production and downstream processes. Bioresour. Technol..

[59] Ma M., Lu B., Irshad S., Chen H., Chu Y., Han D., Hu Q. (2026). Synergistic heterotrophic-photomixotrophic cultivation enhances fucoxanthin production in *Poterioochromonas malhamensis* (golden microalga). Bioresour. Technol..

[60] Guieysse B., Plouviez M. (2024). Microalgae cultivation: Closing the yield gap from laboratory to field scale. Front. Bioeng. Biotechnol..

[61] Khaw Y., Yusoff F., Tan H., Mazli N., Nazarudin M., Shaharuddin N., Omar A., Takahashi K. (2022). Fucoxanthin production of microalgae under different culture factors: A systematic review. Mar. Drugs.

[62] Kanazawa K., Ozaki Y., Hashimoto T., Das S., Matsushita S., Hirano M., Okada T., Komoto A., Mori N., Nakatsuka M. (2008). Commercial-scale preparation of biofunctional fucoxanthin from waste parts of brown sea algae *Laminalia japonica*. Food Sci. Technol. Res..

[63] Derwenskus F., Weickert S., Lewandowski I., Schmid-Staiger U., Hirth T. (2020). Economic evaluation of up- and downstream scenarios for the co-production of fucoxanthin and eicosapentaenoic acid with *P. tricornutum* using flat-panel airlift photobioreactors with artificial light. Algal Res.-Biomass Biofuels Bioprod..

[64] Daniel S., Stefan Z., Gianluca D.A., Maier U.G. (2018). Optimizing CRISPR/Cas9 for the diatom *Phaeodactylum tricornutum*. Front. Plant Sci..

[65] Slattery S.S., Diamond A., Wang H., Therrien J.A., Lant J.T., Jazey T., Lee K., Klassen Z., Desgagné-Penix I., Karas B.J. (2018). An expanded plasmid-based genetic toolbox enables Cas9 genome editing and stable maintenance of syntheticpathways in *Phaeodactylum tricornutum*. ACS Synth. Biol..

[66] Su M., Bastiaens L., Verspreet J., Hayes M. (2023). Applications of microalgae in foods, pharma and feeds and their use as fertilizers and biostimulants: Legislation and regulatory aspects for consideration. Foods.

